# The Impact of Same-Level or Near Peer Assisted Learning Program (NPAP) on Academic Performance of Students in CMH Lahore, Pakistan

**DOI:** 10.12669/pjms.41.3.10968

**Published:** 2025-03

**Authors:** Ayub Ahmad Khan, Shazia Tufail, Mustafa Ayub Khan, Asim Raza

**Affiliations:** 1 Ayub Ahmad Khan, MCPS (ENT), FCPS (ENT), MCPS (HPE) Professor and Head of ENT Department and Medical educationist, CMH Lahore Medical College and Institute of Dentistry, Lahore, Pakistan; 2 Shazia Tufail, MCPS, FCPS, MHPE Associate Professor, Gynae and Obstetrics, CMH Lahore Medical College and Institute of Dentistry, Lahore, Pakistan; 3 Mustafa Ayub Khan, CMHLMC & IOD Undergraduate Student, School of Health Professional Education, CMH Lahore Medical College and Institute of Dentistry, Lahore, Pakistan; 4 Asim Raza Associate Professor (Biostatistics), School of Allied Health Sciences, CMH Lahore Medical College and Institute of Dentistry, Lahore, Pakistan

**Keywords:** Education, Medical, Undergraduate, Otolaryngology, Academic Performance, Quality Improvement, Learning, Near Peer Assisted learning

## Abstract

**Objectives:**

To determine the improvement in academic performance of undergraduate medical students by comparing pre- and post- Near Peer Assisted Learning programs (NPAP) academic scores in the subject of Otorhinolaryngology.

**Methods:**

Our study was a quasi-experimental pretest-posttest study and it was carried out at CMH Lahore Medical College, Lahore during the 2022-2023 academic year. We worked with 72 fourth-year MBBS students, 24 students took on the role of NPAL tutors, while 48 were tutees. The NPAP included regular sessions that emphasized crucial Otorhinolaryngology topics. We gauged academic performance by comparing pretest scores from the 2nd Module Examination with posttest scores from the Send Up and Annual Examinations. Our data analysis, done through IBM SPSS Statistics, focused on both overall and gender-based performance improvements.

**Results:**

Our study demonstrated that NPAP significantly improved academic scores in Otorhinolaryngology, with NPAL tutors showing higher overall improvements in scores. Gender based comparison showed that in both groups of NPAL Tutors and NPAL Tutees, female students scored slightly better than males. The P-value less than 0.001 validated the improvement in academic scores of both NPAP groups of Tutors and Tutees displaying the effectiveness of NPAP as an educational intervention at undergraduate level in the subject of Otorhinolaryngology.

**Conclusions:**

NPAP has shown significant impact on improvement of Academic Performance in undergraduate medical students in the subject of Otorhinolatngology at CMH Lahore Medical College.

## INTRODUCTION

The search for innovative learning strategies in medical education has led to growing interest in Peer Assisted Learning (PAL) program. These initiatives in medical education leverage peer interactions to foster deeper understanding and improved academic outcomes, making them a popular choice in higher education.^1^,^2^

The peer assisted learning (PAL) technique is grounded in Vygotsky’s social constructivist theory, which emphasizes the importance of social interaction in cognitive development.^3^ According to this theory, learning is inherently a social process, and peers within each other’s Zone of Proximal Development (ZPD) can effectively support one another’s learning.^4^ Despite the strong theoretical underpinnings, empirical evidence on the effectiveness of NPAP in improving academic performance remains mixed.^5^ While some studies suggest that NPAL can significantly enhance academic outcomes, others report minimal or no impact, highlighting the need for further research to elucidate the conditions under which NPAL is most effective.^6^

The existing literature on PAL has largely focused on cross-level PAL, where senior students tutor junior peers, often overlooking the potential of same-level PAL. Cross-level PAL has been shown to improve academic performance and develop key skills such as critical thinking and communication.^7^ However, same-level PAL or Near Peer Assisted Learning (NPAL), which encourages collaborative learning among peers at the same academic level, has the potential to create a more equitable and supportive learning environment.

This approach allows students to engage more freely, share knowledge, and develop a deeper understanding of the material without the hierarchical barriers often present in cross-level interactions. Near Peer Assisted Learning Programs (NPAP) approach posits that peers, sharing similar academic experiences and challenges, are uniquely positioned to facilitate learning and address each other’s needs effectively and has emerged as a particularly promising model.^8^

There is a significant gap in the literature regarding the impact of NPAL on academic performance, particularly in the context of medical education.^9^ Medical education presents unique challenges, including the need for students to acquire a vast amount of knowledge in a short period and develop critical clinical skills. Given these demands, innovative educational strategies like PAL are crucial for supporting student success.^10^ However, most studies on PAL in medical education have focused on cross-level interactions, with limited attention given to same-level PAL.^8^

The rationale of our study drives from the need to explore alternative supplemental educational interventions that aims to fill a critical gap in the literature by examining the impact of same-level PAL or Near Peer Assisted Learning on academic performance in a medical education setting. By leveraging a comprehensive dataset and employing rigorous statistical analyses, the study seeks to provide valuable insights into the effectiveness of same-level peer or Near Peer Assisted Learning interventions and contribute to the ongoing efforts to improve educational outcomes for medical students.

## METHODS

Our study used a pretest-posttest design for this quasi-experimental study to evaluate the effectiveness of Near Peer Assisted Learning (NPAL) on the academic performance of 4th-year MBBS students. The study was conducted at CMH Lahore Medical College, Lahore, Pakistan, during the academic year 2022-2023.Sample size included 72 fourth-year MBBS students who volunteered for this program. Among these, 24 students were selected as NPAL tutors, while the remaining 48 students were selected as NPAL tutees based on their academic performance in the 2nd Module Examination. Informed consent was obtained from all participants and confidentiality and anonymity of the participants were maintained throughout the study.

### The brief summary of NPAL program was as follows: Orientation:

A lecture was arranged for fourth-year students to explain the rationale of the study.

### Grouping of the class:

Identification and grouping of whole class into five strata from very weak to very bright students based on Academic performance in module 1 and module 2 assessments with bench mark of 50 percent and attendance with bench mark of 75 percent. This grouping was kept confidential and blinded from the students to avoid any issues of discrimination amongst the students (Table-I).

**Table-I:** Grouping table based on attendance and internal assessment.

Grading	Attendance	IA	Grading
Very Bright student	>75%	>85%	Very Bright student
Bright student	>75%	btw 50 to 84%	Bright student
Borderline	60 to 75%	Btw 40 to 50	Borderline
Weak student	50 to 60%	Btw 30 to 40	Weak student

In our Pilot Project, 12 groups were made, each group consisting of 2 NPAL Tutors and 4 NPAL tutees and type of intervention was Near Peer Tutoring with Ratio of 1:4. The top 24 students belonging to very bright category with high academic achievement were selected as NPAL tutors with voluntary induction. The lower 48 students belonging to very weak and weak strata with poor academic performance were selected as NPAL Tutees with voluntary induction.

### NPAL Tutor training:

Formal training sessions in the form of 08 workshops was conducted by the dedicated NPAL faculty team for the NPAL tutors. The topics of the workshops included general teaching principles like psychology behind teaching and learning, principles of adult learning, NPAL techniques, how to write learning objectives, modes of information transfer, learning styles, assessment techniques and feedback techniques. Pre and post workshops OSTE for NPAL Tutors to check for prior knowledge and preparedness for NPAL Tutoring.

### NPAL sessions:

The course content was the prescribed Otorhinolaryngology curriculum of NUMS. The focus of intervention included two primary areas, course content component and preparing for assessment component. The NPAL intervention included lecture-based tutorials, small group discussions, assessment of course content covered. The tutors prepared their own lesson plan with the help of NPAL faculty team for the NPAL interactions including learning outcomes, format, sequence of activity and room layout. The NPAL tutors were encouraged to reflect on their teaching experience through learning plans, logbooks and diaries. Two NPAL sessions were conducted in each academic week from 1^st^ July to 31^st^ August 2022 during the Self-Directed Learning slots allocated to ENT as an Extracurricular activity. The NPAL Sessions were scheduled on Tuesdays and Thursdays or any two days of the week as the groups mutually agree with a duration of two hours at the time of their own choice. The mode of interaction was the choice of NPAL Tutors according to their own lesson plan. Attendance was uploaded for each session on the google document attached to the WhatsApp group for the NPAL Tutors. Each NPAL session was concluded with filling of feedback forms and NPAL tutor providing formative feedback to each NPAL tutee. Each feedback and evaluation form was filled up by every NPAL tutor and every Tutee at the end of each NPAL session.

### Ethical Approval:

The study was approved by the Institutional Review Board (IRB) of CMH Lahore Medical College vide letter no Case# 752/ERC/CMH/LMC dated 12/04/2023.

### Inclusion Criteria:

4th-year MBBS students enrolled in the Otorhinolaryngology course, willingness to participate in the NPAL program.

### Exclusion Criteria:

Students who did not consent to Participate, students who did not regularly attend the NPAL Tutor workshops, students who were not regularly attending the NPAL sessions. The Intervention in our study was Near Peer Assisted Learning (NPAL) conducted on a structured program. The NPAL program involved selected students (NPAL tutors) conducting regular study sessions with their peers (NPAL tutees) throughout the semester. These sessions focused on key topics within the Otorhinolaryngology curriculum, and NPAL tutors provided academic support, clarification of concepts, and exam preparation strategies.

### The data was collected according to the following data points:

### Pretest:

Academic performance data was collected at the beginning of the study from the 2nd Module Examination scores.

### Posttest:

Academic performance data was collected at the end of the study from the Send Up and Annual Examination scores. The primary outcome measure was the change in academic performance, assessed by comparing the pretest (2nd Module Examination scores) and posttest (Send Up and Annual Examination scores) results in percentages along with gender-based differences and differences in performance of NPAL Tutors versus NPAL Tutees.

Data analysis in the study was performed using IBM SPSS Statistics software version 26.00. For the descriptive statistics, mean and standard deviation were calculated for ages, median and inter quartile range were calculated for 2^nd^ modules, sendup and annual examination marks because data was skewed Whereas, frequencies and percentages were calculated for qualitative variable like gender. The Chi-square test was applied to compare the frequencies and percentages. Shapiro-Wilk test was applied to test the normality of data. For the comparison of exam scores for 2^nd^ modules, sendup and annual examination; Friedman test was applied whereas Bonferroni Correction was done as multiple comparison test.

## RESULTS

### The results of our study are elaborated according to objectives as following:

***Evaluation of Overall Academic Impact of NPAL on academic performance of students:*** The NPAL tutees examination (2^nd^ module, sendup and annual) scores in percentages were compared by non-parametric Friedman test, median scores percentages were found 38.50, 58.00 and 74.50 that were statistically significant with p-value ≤0.05, show in Table-II. Bonferroni correction adjustments were also made for Multiple comparison test that were also statistically significant with p-value ≤0.05 in Table-III. It means that a significant improvement was found in tutees Near Peer Assisted Learning programs (NPAP) academic scores in the subject of Otorhinolaryngology.

**Table-II:** Comparison of Exams scores in percentages of NPAL tutees in percentages, (n= 48).

Examination	Median (IQR=Q3-Q1)	Mean Rank	Friedman Test	P-value
2nd Module Scores (%)	38.50 (43.00-38.00)	1.05	81.611	<0.001*
Send Up Scores (%)	58.00(62.50-52.25)	1.99		
Annual Exam Scores (%)	74.50(78.00-71.00)	2.97		

“*” indicates the statistical significance with p-value ≤0.05.

**Table-III:** Multiple comparisons of NPAL tutee’s exam scores in percentages.

Sample 1-Sample 2	Test Statistic	Std. Error	Std. Test Statistic	P-Value	Adj. P-value
2nd Module - Send Up	-0.943	0.213	-4.424	<0.001*	<0.001*
2nd Module - Annual Exam	-1.920	0.213	-9.008	<0.001*	<0.001*
Send Up - Annual Exam	-0.977	0.213	-4.584	<0.001*	<0.001*

“*” indicates the statistical significance with p-value ≤0.05. “a “Significance values have been adjusted by the Bonferroni correction for multiple tests.

The NPAL tutors’ examination (2^nd^ module, sendup and annual) scores in percentages were compared by non-parametric Friedman test, median scores percentages were found 59.50, 67.50 and 86.00 that were statistically significant with p-value ≤0.05, show in Table-IV. Bonferroni correction adjustments were also made for Multiple comparison test; 2^nd^ Module to Annual Exam and Send up to Annual exam that were statistically significant with p-value ≤0.05 in Table-V. It means that a significant improvement was found in tutors Near Peer Assisted Learning programs (NPAP) academic scores in the subject of Otorhinolaryngology.

**Table-IV:** Compare the Exam scores of NPAL tutors in percentages, (n=24).

Examination	Median (IQR=Q3-Q1)	Mean Rank	Friedman Test	P-value
2nd Module Scores (%)	59.50(66.25-55.25)	1.23	37.12	<0.001*
Send Up Scores (%)	67.50(71.00-62.00)	1.77		
Annual Exam Scores (%)	86.00(88.25-84.00)	3.00		

“*” indicates the statistical significance with p-value ≤0.05.

**Table-V:** Multiple comparisons of NPAL tutor’s exam scores in percentages.

Sample 1-Sample 2	Test Statistic	Std. Error	Std. Test Statistic	P-Value	Adj. P-value^a^
2nd Module - Send Up	-0.545	0.302	-1.809	0.070	0.211
2nd Module - Annual Exam	-1.773	0.302	-5.879	<0.001*	<0.001*
Send Up - Annual Exam	-1.227	0.302	-4.070	<0.001*	<0.001*

“*” indicates the statistical significance with p-value ≤0.05. “a” Significance values have been adjusted by the Bonferroni correction for multiple tests.

Non-parametric Mann Whitney U test was applied for between groups (NPAL tutors and tutees) comparison of average exam (2^nd^ module, sendup and annual) scores in percentages. Statistically significant change was found with p-value ≤0.05 between NPAL tutors and tutees average scores in 2^nd^ module, sendup and annual exams. Improvement in average scores was more among NPAL tutors than tutees that are shown in Table-VI.

**Table-VI:** Between groups (NPAL tutors and tutees) comparison of exam scores in percentages.

Examination	Median (IQR=Q3-Q1)		Tutors	Tutees	Mann-Whitney U test	P-value

	Tutor, (n=22)	Tutees, (n=44)	Mean Rank			
2nd Module Scores (%)	59.50(66.25-55.25)	38.50(43.00-38.50)	54.70	22.90	17.5	<0.001*
Send Up Scores (%)	67.50(71.00-62.00)	58.00(62.50-52.25)	49.48	25.51	132.5	<0.001*
Annual Exam Scores (%)	86.00(88.25-84.00)	74.50(78.00-71.00)	54.66	22.92	18.5	<0.001*

“*” indicates the statistical significance with p-value ≤0.05.

The overall academic performance of 4th-year MBBS students in Otorhinolaryngology showed significant improvement after participating in NPAL programs. The mean percentage scores for NPAL tutees increased from the 2^nd^ Module to the Annual Exam, as did those for NPAL tutors shown in Table-II and Table-IV respectively. While both groups benefited from the NPAL program, tutors exhibited a slightly higher improvement in academic performance compared to tutees shown in Table-VI.

### Evaluation and analysis of gender-based differences in the improvement in academic performance of students participating as NPAL tutors and tutees:

The gender distribution of NPAL Tutees included: Male: 20 (45.5%) and Female: 24 (54.5%) while the gender distribution of the NPAL Tutors included: Male: 10 (45.5%) and Female: 12 (54.5%). The comparison of improvement in academic performance as a result of NPAL intervention gender-based is shown Table-VII, with females performing better in both groups of NPAL Tutees and NPAL tutors. Fig-I and Fig-2.

**Table-VII:** Gender based improvement in academic performance.

Group	Gender	2nd Module (%)	Send Up (%)	Annual Exam (%)
NPAL Tutees	Male	40.1	57.2	72.9
	Female	36.5	58.7	75.4
NPAL Tutors	Male	58.5	65.0	85.0
	Female	60.5	69.5	87.0

**Fig.1:**
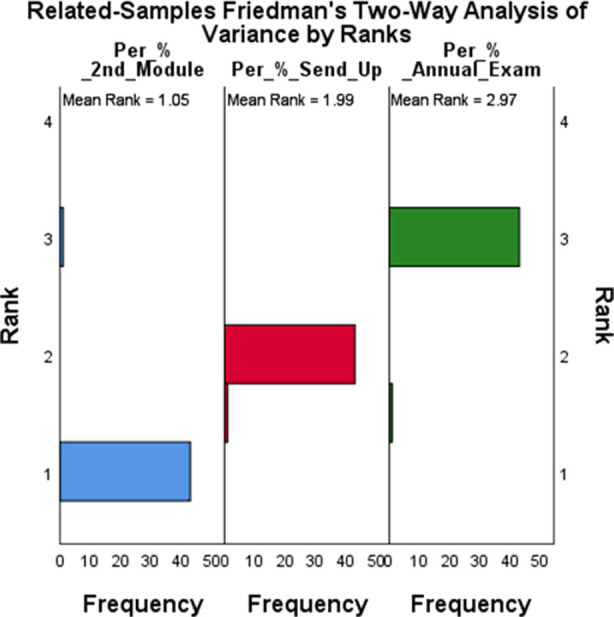
Distribution of academic scores of NPAP Tutees.

**Fig.2:**
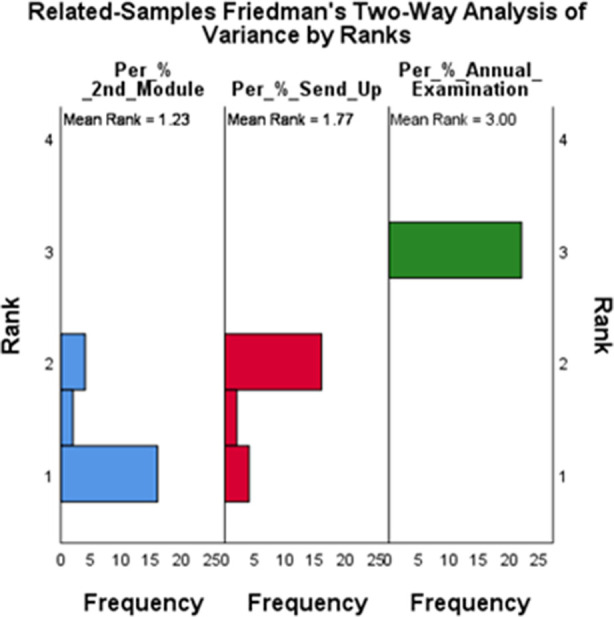
Distribution of academic scores of NPAP Tutors.

### Data-driven insights for enhancement of NPAL programs based on the observed academic performance outcomes:

Both NPAL tutees and tutors exhibited significant improvements from the 2nd Module to the Annual Examination as shown in Table-II, III, IV and V. NPAL tutors performed better than NPAL tutees in all examination, suggesting a positive correlation between teaching and academic performance as shown in Table-VI. Female students generally showed higher median scores compared to male students across all exams as shown in Table-VII.

## DISCUSSION

Our study demonstrated that NPAP significantly improved academic scores in Otorhinolaryngology, with NPAL tutors showing higher overall improvements in scores. Gender based comparison showed that in both groups of NPAL Tutors and NPAL Tutees, female students scored slightly better than males. The P-value less than 0.001 validated the improvement in academic scores of both NPAP groups of Tutors and Tutees displaying the effectiveness of NPAP as an educational intervention at undergraduate level in the subject of Otorhinolaryngology. Our research shows that both NPAL tutees and tutors demonstrated substantial academic gains. These improvements highlight the effectiveness of NPAL in reinforcing students’ understanding and retention of subject matter, aligning with previous research that underscores the benefits of peer-assisted learning in medical education.^11^,^12^ Another study by Hernández et al. identified that PAL led to benefits like increase in tutors’ confidence and knowledge, better student comprehension and a more comfortable learning environment in comparison with that established by academic tutors.^13^

Our study aligns with existing research in demonstrating that NPAL tutors consistently outperformed tutees, which could be attributed to the ‘teaching effect’ where tutors reinforce their own knowledge through teaching peers.^14^ This reinforces the dual benefit of NPAL, providing educational value to both tutors and tutees. Gender analysis in our study revealed that female students generally outperformed their male counterparts across all exam stages. Similarly, female NPAL tutors showed higher scores than male tutors. These findings suggest that female students might derive greater benefit from NPAL programs, potentially due to differences in learning styles or engagement levels. This is consistent with research that suggests gender can influence academic outcomes and learning preferences.^15^

The statistical validation was carried out using the Friedman test which confirmed the statistical significance of the observed academic improvements. For both NPAP tutees and tutors, significant differences were found between the 2nd Module, Send Up, and Annual Exam scores (p < 0.001). This robust statistical evidence supports the effectiveness of NPAL programs in enhancing academic performance. This finding is consistent with the findings of the studies in the literature.^16^ The importance of our study originates from the need to explore alternative supplemental educational interventions that can enhance academic performance in undergraduate medical education specifically focusing on Otorhinolaryngology and address the existing gaps in literature in terms of improved academic outcomes that vary across disciplines.^17^

Also, our study provides a comprehensive evaluation of the effectiveness of same-level PAL or Near Peer Assisted Learning.^18^,^19^ Notably, our study addresses the current gap in literature, which has predominantly focused on cross-level PAL interventions, thus underscoring the advantages of same-level PAL in enhancing understanding without hierarchical constraints.^20^ Additionally, our study focuses on the gender-based differences in academic performance, an area that has received limited attention in previous studies.^21^

Furthermore, the findings of our study are expected to have significant evidence based implications for the implementation of NPAL programs in medical education and provide actionable recommendations for educators and policymakers.^22^ Our study would also address the methodological limitations of previous research by using a pretest-posttest design, which allows for a more accurate assessment of the intervention’s impact on academic performance 22 and this brings to light the main strength of our study also. Larger number of students involved in NPAL and continuous NPAP are required to be run by institutions with in department qualitative research to further study the advantages and pitfalls of NPAP.

### Limitations:

It included relatively small sample size, particularly for NPAL tutors, may limit the generalizability of the results. The study was conducted at a single medical college, which may not reflect the broader population of medical students. Finally the study did not control the potential confounding factors such as individual student motivation, prior knowledge, and external support systems.

## CONCLUSION

Our study concluded that NPAP had statistically shown significant impact on improvement of Academic Performance in undergraduate students in the subject of Otorhinolatngology at CMH Lahore Medical College, underscoring the effectiveness of NPAP as an educational intervention. Both NPAP tutees and tutors benefitted from the program, with NPAP tutors showing higher overall improvements. Gender differences indicated that female students out performed their male counter parts in both the groups. Our findings provide compelling evidence for the continued implementation and expansion of NPAL programs in medical education.

### Recommendations::

- Include NPAP as a supplemental elective part of medical curriculum especially for weak students.
- Provide additional support and training for NPAP tutors to maximize their effectiveness and the benefits they derive from the program.
- Develop and implement strategies that cater to different learning styles and needs of male and female students to optimize the benefits of NPAP programs.
- Investigate the long-term impacts of NPAPL on both academic performance and professional development of medical students by conducting a Longitudinal Research.

### Authors’ Contribution:

**AAK:** Conceived, designed and did statistical analysis & final editing of manuscript, is responsible for integrity of research.

**ST, MAK and AR:** Did data collection, critical analysis and manuscript writing.

**MAK:** Critical review and editing of manuscript.

**AR:** Helped with the statistical analysis..

All authors have read, approved final version and are accountable for the integrity of the study.
